# Protocol for Imaging and Analysis of Mouse Tumor Models with CUBIC Tissue Clearing

**DOI:** 10.1016/j.xpro.2020.100191

**Published:** 2020-11-26

**Authors:** Kei Takahashi, Shimpei I. Kubota, Shogo Ehata, Hiroki R. Ueda, Kohei Miyazono

**Affiliations:** 1 Department of Molecular Pathology, Graduate School of Medicine, The University of Tokyo, 7-3-1, Hongo, Bunkyo-ku, Tokyo 113-0033, Japan; 2 Department of Systems Pharmacology, Graduate School of Medicine, The University of Tokyo, 7-3-1, Hongo, Bunkyo-ku, Tokyo 113-0033, Japan; 3 Laboratory for Synthetic Biology, RIKEN Center for Biosystems Dynamics Research, 1-3, Yamadaoka, Suita, Osaka 565-0871, Japan

**Keywords:** Cancer, Microscopy, Model Organisms

## Abstract

Tissue-clearing technologies have developed rapidly in the past decade, especially for use in neuroscience research. We previously reported that CUBIC, which is one tissue-clearing method, is useful for applications in cancer research. CUBIC cancer analysis can be used to detect cancer metastasis with single-cell resolution at whole mouse body/organ level. This approach can also analyze the tumor characteristics with high-quality 3D images. Here, we describe a detailed CUBIC cancer protocol from tissue clearing, capturing 3D images and post-immunohistochemistry.

For complete details on the use and execution of this protocol, please refer to Kubota et al. (2017).

## Highlights

• Detailed protocol for making mouse organs transparent using CUBIC reagents
• Method to capture 2D microscope images, convert to 3D, and analyze images
• Description of immunohistochemistry with CUBIC-cleared mouse organs

## Graphical Abstract

**Figure:**
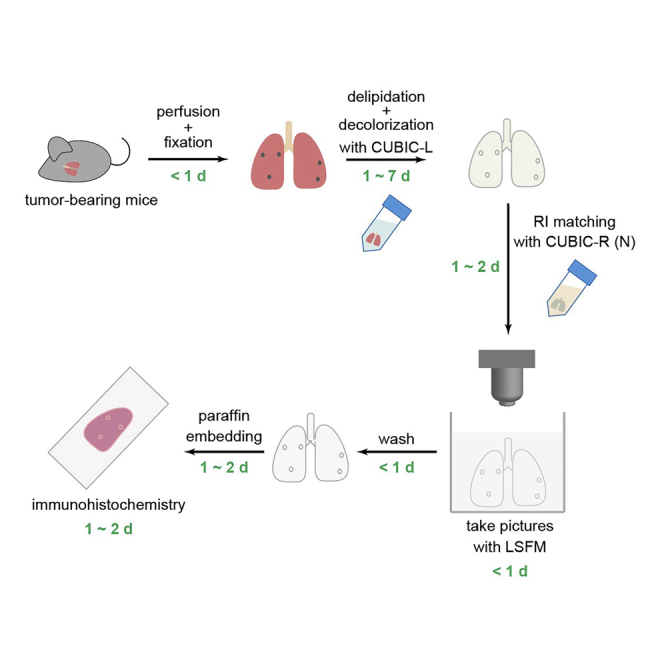

## Before You Begin

### Establishment of Tumor-Bearing Mouse Models

**Timing: 1 day to several months**

1. Establishment of cancer metastasis mouse models using cancer cells stably expressing fluorescence proteins.
  a. Anesthetize mice with isoflurane if necessary.
  b. Inoculate cancer cells stably expressing fluorescence proteins (i.e., mCherry, tdTomato) or fluorescent dye (i.e., fluorescein isothiocyanate) into mice, intravenously, orthotopically, subcutaneously, or intracardially, dependent upon experiments.
2. ***Optional:*** When cancer cells are also expressing firefly luciferase gene, monitor tumor progression using *in vivo* bioluminescence imaging.

**Table:** Representative inoculation mouse models

How to inject	Metastatic organs (typically)	Injection volume (typically)
Intravenously (iv)	lung, (liver)	100–500 μL
Intracardially (ic)	bone, brain	200 μL
Subcutaneously (sc)	lung, lymph node	50–100 μL

### Preparation of Reagents

**Timing: 1 h**

3. CUBIC-L reagent (100 g) (pH < 11)
  a. Add 10 g of polyethylene glycol mono-p-isooctylphenyl ether/TritonX-100 into a 100 mL tube or a beaker.
  b. Add 10 g of N-butyldiethanolamine (pH 10–11).
  c. Add 80 g of ultra-pure water and shake gently (with a stirrer).
  d. Store in a sealed container at 18°C–26°C.
4. CUBIC-R (N) reagent (amber-colored) (100 g) (Refractive index (RI) = 1.52, Temperature = 25°C)
  a. Add 30 g of nicotinamide into a 100 mL tube or a beaker.
  b. Add 45 g of 2,3-dimethyl-1-phenyl-5-pyrazolone/antipyrine.
  c. Add 25 g of ultra-pure water and mix.
  d. Use microwave or heating stirrer up to around 70°C to dissolve them and shake gently. Chill them to 18°C–26°C.
  e. ***Optional:*** Measure the RI index using RI measurement equipment and adjust to 1.52 by adding ultra-pure water.
  f. Store at 18°C–26°C in a sealed container and protect from the light.
5. 3D staining buffer (100 g)
  a. Add 25 g of casein (final 0.25%) into a 100 mL tube.
  b. Add 2.5 mL of 20% Triton X-100 (final 0.5%).
  c. Add 100 ul sodium azide (final 0.01%).
  d. Add PBS up to 100 g and shake gently.
  e. Store at 4°C.
6. 4% paraformaldehyde (PFA) (100 g) (pH 7–8)
  a. Prepare 50–60 mL ultra-pure water warmed up to 60°C–70°C by microwave or heating stirrer.
  b. Add 4 g of PFA into warmed water and mix them with a stirrer.
  c. Add 0.1 N NaOH to dissolve PFA by changing pH.
  d. Add 4 mL of PBS (25×) and mess up to 100 g by ultra-pure water. Adjust final pH to 7.2–7.4 with 0.1 N NaOH.
  e. Store at −20°C.

**CRITICAL:** Appropriate pH of each reagent is important and high or low pH can become a problem for quenching. Since CUBIC-R (N) contains near-limiting amounts of nicotinamide and 2,3-dimethyl-1-phenyl-5-pyrazolone, it might precipitate at low room temperature. CUBIC-L and CUBIC-R (N) are diluted by half with ultra-pure water and used as 50% CUBIC-L and 50% CUBIC-R (N).

***Note:*** CUBIC-L reagent can be mixed by just shaking without warming. Pre-mixed and quality-controlled CUBIC-L is also commercially available. In addition, CUBIC-R (N) can be prepared as described, but a pH-adjusted and quality-controlled reagent CUBIC-R+ can be also purchased. If using microscope is not compatible to RI=1.52, add ultra-pure water to CUBIC-R (N) to decrease the value of RI, which is compatible to your microscope.

### Preparation of Reagents Required for Image Capture

**Timing: 15 min**

7. Observation oil (RI = 1.52, Temperature = 25°C)
  a. Add 60.2 g of HIVAC F-4 (RI = 1.555, 25°C) into a 100 mL tube.
  b. Add 39.8 g of mineral oil (RI = 1.467, 25°C) and shake gently.
  c. Measure the RI index using RI measurement equipment and adjust by adding HIVAC-F4 or mineral oil to 1.52.

**CRITICAL:** For taking high-quality pictures, it is important that the refractive index of CUBIC-R (N) and observation oil match.

***Note:*** Pre-mixed and quality-controlled observation oil (Mounting solution for CUBIC-R+) is alternatively purchased.

### Preparation of Reagents for Immunohistochemistry

**Timing: 15 min**

8. Endogenous peroxidase blocking solution
  a. Mix 270 mL methanol and 30 mL hydrogen peroxide.
  b. Store at 18°C–26°C in sealed container in the dark.
9. Antigen retrieval solution
  a. Dilute 30 mL Universal HIER antigen retrieval reagent (10×) with 270 mL ultra-pure water.
  b. Store at 18°C–26°C in sealed container.

***Note:*** Although it basically depends on the manufactural protocols, the antigen retrieval solution can be usually used for 4–5 times.

## Key Resources Table

**Table:** 

Reagent or Resource	Source	Identifier		
**Antibodies**				

Monoclonal anti-actin, α-smooth muscle-FITC antibody	Sigma-Aldrich			Cat#F3777
RedDot2	Biotium Inc			Cat#40061
Anti-E-cadherin (24E10) antibody	Cell Signaling Technology			Cat#3195
**Chemicals, Peptides, and Recombinant Proteins**				

Hydrogen peroxide	Wako			Cat#081-04215
Methanol	Nacalai Tesque			Cat#21915-93
Universal HIER antigen retrieval reagent (10×)	Abcam			Cat#ab208572
Blocking One	Nacalai Tesque			Cat#03953-95
VECTASTAIN Elite ABC Rabbit IgG Kit	VECTOR Laboratories			Cat#PK-6101
ImmPACT DAB	VECTOR Laboratories			Cat#SK-4105

Polyethylene glycol mono-p-isooctylphenyl ether/TritonX-100	Nacalai Tesque	Cat#12967-45		
N-Butyldiethanolamine	Tokyo Chemical Industry	Cat#B0725		
CUBIC-L (for animals)	Tokyo Chemical Industry	Cat#T3740		
2,3-Dimethyl-1-phenyl-5-pyrazolone/antipyrine	Tokyo Chemical Industry	Cat#D1876		
Nicotinamide	Tokyo Chemical Industry	Cat#N0078		
CUBIC-R+ (for animals)	Tokyo Chemical Industry	Cat#T3741		
HIVAC-F4	Shin-Etsu Chemical			
Mineral oil	Sigma-Aldrich	Cat#M8410		
Mounting solution (for CUBIC-R+)	Tokyo Chemical Industry	Cat#M3294		
Blocker Casein in PBS	Thermo Fisher Scientific	Cat#37528		
Sodium azide	Nacalai Tesque	Cat#31208-82		
Paraformaldehyde	Wako	Cat#162-16065		
Osteosoft	Merk Millipore	Cat#101728		
PBS (phosphate-buffered saline) (pH 7.4)	n/a	n/a		
TBS (Tris-buffered saline)	n/a	n/a		
PB (phosphate buffer)	n/a	n/a		
Pentobarbital	Kyoritsu Seiyaku	n/a		
Isoflurane	FUJIFILM Wako Pure Chemical Corporation	Cat#099-06571		

**Software**				

Imaris software	https://imaris.oxinst.com/		n/a	
ImageJ FIJI	https://imagej.nih.gov/ij/ https://fiji.sc/		n/a	

**Other**				

Light sheet fluorescence microscope (LSFM)	Olympus, custom-made	n/a		
Confocal microscope	Olympus	FLOVIEW FV1200		
*In vivo* imaging system	Berthold Technologies PerkinElmer	Night OWL IVIS (Spectrum, Lumina)		
Three-way stopcock	TERUMO	Cat#TS-TR2K		
30 mL syringe	Nipro	Cat#08-853		
Shaker	n/a	n/a		
RI measurement equipment	ATAGO	Cat#DR-A1		
Paraffin-embedded block manufacturing equipment	n/a	n/a

***Alternatives:*** Instead of Imaris software, images can be analyzed with ImageJ or FIJI, BigStitcher, NIS-Elements.

***Alternatives:*** Rice steamer or automated device for antigen retrieval like LAB Vision PT Module (Thermo Scientific) can be also used instead of autoclave machine. (related to step 20)

## Step-By-Step Method Details

### Tissue Clearing with CUBIC Reagents

**Timing: 3–14 days**

This step describes how to prepare mouse samples and how to clear the organs.

1. Sacrifice tumor-bearing mice by overdose of pentobarbital (> 100 mg/kg) or isoflurane.
2. Perfuse PBS and PFA.
  a. Make an incision in the right atrial appendage of heart.
  b. Perfuse 20–25 mL PBS via left ventricle of the heart using syringe with 23G needle.
  c. After PBS perfusion, change solution from PBS to 4% PFA by a three-way cock.
  d. Perfuse 20–25 mL 4% PFA.
3. Excise organs.

***Note:*** Coagulation might be seen in the spleen after excision. The coagulated blood is very difficult to clear.

**CRITICAL:** From step 4 to step 12, samples should be protected from the light.

4. Immerse excised organs (samples) into 4% PFA and shake gently at 4°C for overnight (12–16 h).
5. Wash samples with PBS to remove 4% PFA.
  a. Exchange solution from PFA to PBS and shake gently for more than 2 h.
  b. Exchange PBS more than 2 times (total wash > 6 h).
6. Exchange solution from PBS to 50% CUBIC-L and shake gently at 37°C (50–100 rpm).
7. After over 6 h of incubation, exchange solution with 100% CUBIC-L, shake gently at 37°C for 1–7 days (until the exchanged CUBIC-L solution is not colored).

**Figure 1:**
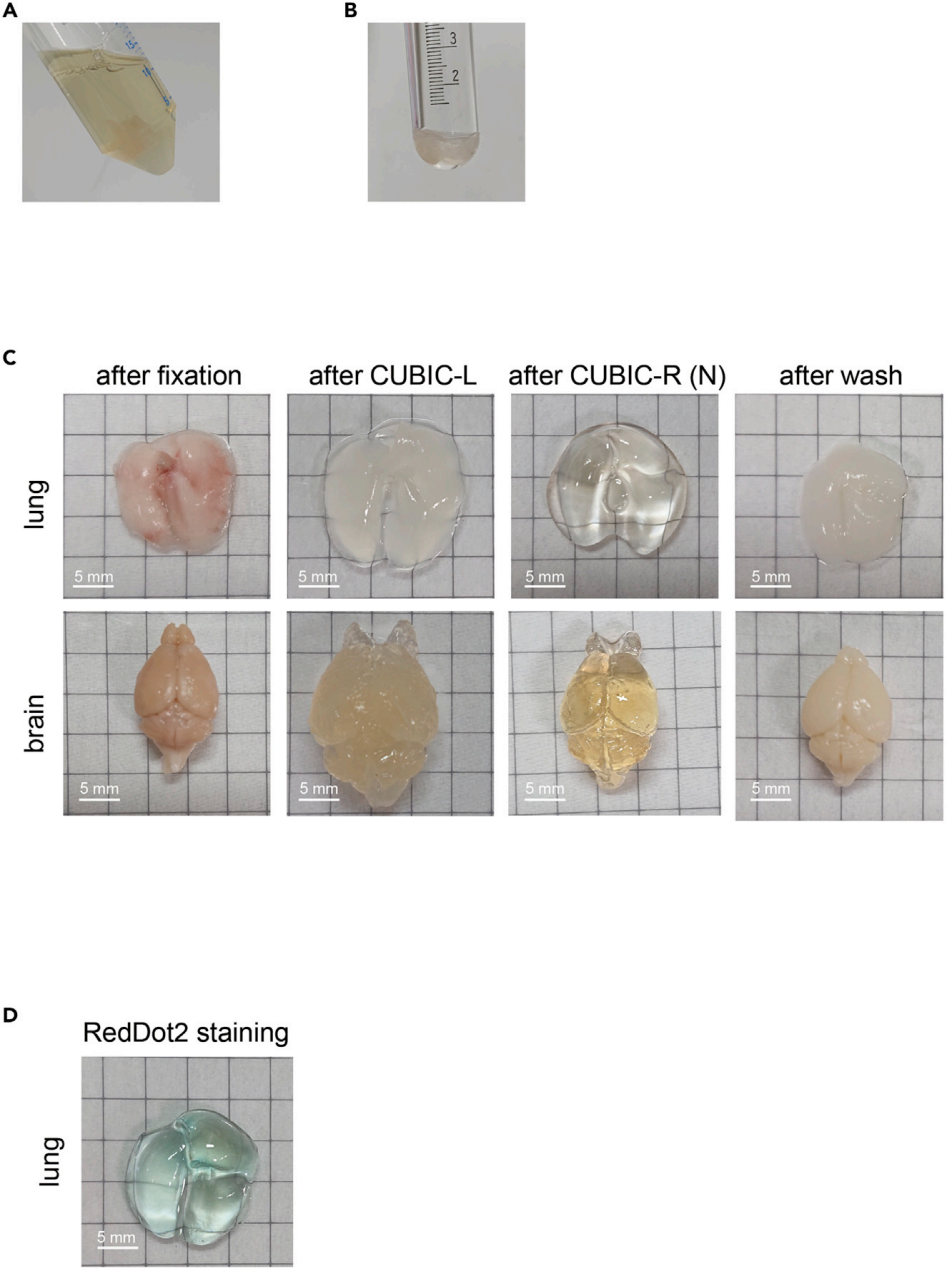
Whole Mouse Organ Clearing with CUBIC Reagents (A) The colored 50% CUBIC-L after gently shaking at 37°C overnight (after step 6). (B) The 3D staining of intestine with flat bottom containers (step 9). (C) The images of lung and brain at each step (most left, after fixation; second left, after delipidation and decolorization with CUBIC-L; second right, after RI adjustment with CUBIC-R (N); most right, after PBS wash with transparent organs). (D) The image of transparent lung stained with RedDot2 (after steps 12 and 13).

**CRITICAL:** In steps 6 and 7 (delipidation and decolorization process), the solution will be colored as shown in Figure 1A. Exchange CUBIC-L until the solution is no longer colored. Moreover, CUBIC-L might damage some kinds of tubes. Exchange tubes if it happens.

8. Wash samples with PBS to remove CUBIC-L.
  a. Exchange solution from CUBIC-L to PBS and shake gently for more than 2 h.
  b. Exchange PBS more than 2 times (total wash > 6 h).
9. ***Optional:*** In case of 3D staining (immunostaining including nuclear staining).
  a. Staining with antibody.
    (i) Immerse samples with antibody dilution in staining buffer (1:100–1:400 depending upon antibody). Shake gently at 22°C for 3 days. Protect from the light.
  b. Remove antibody solution and wash in PBS.
    (i) Exchange solution from antibody solution to PBS and shake gently for more than 2 h.
    (ii) Exchange PBS more than 2–4 times (total wash > 6 h).

***Optional:*** Before step 9a, centrifuge diluted antibodies to remove precipitate aggregates (1,000–1,500 × *g* for 15 min). Use supernatant.

**CRITICAL:** Immerse entire organs in staining buffer. Use a container with a round bottom as shown in Figure 1B. We prefer to use fluorescent dye or fluorescence proteins conjugated primary antibodies for uniform deep staining.

10. ***Optional:*** In case of bone samples, decalcification process is needed (Tainaka et al., 2018).
  a. Immerse bone samples in decalcification solution (i.e., osteosoft).
  b. ***Optional:*** Remove air using vacuum desiccator (10–20 in/hg).
  c. Leave them at 18°C–26°C for 5–7 days.
  d. Wash samples with PBS to remove antibody solution.
    (i) Exchange solution from antibody solution to PBS and shake gently for more than 2 h.
    (ii) Exchange PBS more than 2 times (total wash > 6 h).
11. Exchange solution to 50% CUBIC-R (N) and shake gently at 18°C–26°C for more than 6 h. Protect from the light.
12. Exchange solution to 100% CUBIC-R (N) and shake gently at 18°C–26°C for overnight (12–16 h). Protect from the light.

***Note:*** The liver might become white instead of clear in very rare cases. We are looking into the cause of this problem, but we have yet to solve it.

***Optional:*** To avoid movements of organs while acquiring the images by LSFM, organs can be embedded in 2% gel before step 11.

**Table:** Standard amounts of reagents and times of shaking

Organ	Amounts of CUBIC-L and CUBIC-R (N) used	CUBIC-L
Lung	10–20 mL	1–3 days
Brain	20–30 mL	2–4 days
Liver	30–40 mL	5–7 days
Kidney	10–20 mL	2–4 days
Heart	10–20 mL	2–4 days

### Capture 3D Images

**Timing: 1 day**

This step describes how to obtain images of cleared tissue samples with LSFM or confocal microscopy.

13. Take images with light sheet fluorescence microscopy or confocal microscopy.
  a. Wash samples in CUBIC-R (N) with observation oil.
  b. Put samples into a glass container fulfilled with observation oil.
  c. Collect images by light sheet fluorescent microscopy (LSFM) (z = 10 μm step).
14. Record images (TIFF format) and analyze data with Imaris software (see Quantification Analysis).

***Note:*** Using custom-made LSFM, images were captured at 0.63 × objective lens (numerical aperture = 0.15, working distance = 87 mm) with zoom from 1 × to 6.3 × zoom (zoom 1.25 ×; pixel resolution(x): 8.25 μm, pixel resolution(y): 8.25 μm, pixel resolution(z): 10 μm) (zoom 1.6 ×; pixel resolution(x): 6.45 μm, pixel resolution(y): 6.45 μm, pixel resolution(z): 10 μm). All raw image data were collected in a lossless 16-bit TIFF format.

### Post-imaging Immunohistochemistry (DAB)

**Timing: 2–4 days**

This step describes how to prepare samples for immunohistochemistry.

15. Wash samples with PBS.
  a. Put samples into PBS and shake gently for more than 2 h.
  b. Exchange PBS more than 2 times (total wash > 6 h).
16. Embed samples into paraffin using equipment (including dehydration and delipidation process with ethanol and xylene).
17. Make sections using standard histology microtome and mount on glass slide.
18. Deparaffinize slides with xylene and ethanol.
19. Remove endogenous peroxidase.
  a. Immerse slides in endogenous peroxidase blocking solution.
  b. Incubate them at 18°C–26°C for 15–20 min.
  c. Wash with tap water or TBS.
20. Antigen retrieval.
  a. Immerse slides into HIER.
  b. Autoclave them at 121°C for 10 min.
  c. Chill them to 18°C–26°C.
21. Wash with TBS, rinse with water (to prevent salt crystals) and dry.
22. Cover 1st antibody and incubate for 2 h to 16 h in a humidified chamber (to prevent drying out).
23. Wash with TBS.
  a. Exchange solution to TBS and still for 3 min.
  b. Repeat “a” more than 2 times.
24. Add 2nd antibody labeled with biotin and incubate at 40°C for 30 min.
25. Wash with TBS.
  a. Exchange solution to TBS and still for 3 min.
  b. Repeat “a” more than 2 times.
26. Add ABC reagent and incubate at 40°C for 20–30 min.
27. Wash with TBS.
  a. Exchange solution to TBS and still for 3 min.
  b. Repeat “a” more than 2 times.
28. DAB coloring.
  a. Add DAB dilution and incubate for 2–3 min at 18°C–26°C.
  b. Wash with TBS.
29. Stain nucleus with Meyer.
30. Wash with tap water.
31. Dehydration with ethanol and xylene.

32. Mount slides with coverslip using mounting agents (Malinol, Permount, Histoclad, etc).
  ***Note:*** We showed avidin-biotin complex (ABC) method, but we can also apply other direct or indirect immunohistochemistry methods.
  ***Alternatives:*** PBS can be used instead of TBS.
  ***Optional:*** In case of H&E staining, after step 18, stain slides with hematoxylin and eosin.
  ***Optional:*** When making frozen sections with samples after PBS wash (step 14), sucrose exchange process is needed.
  a. Immerse samples in 5% sucrose in PB and shake gently for 30 min to 1 h.
  b. Exchange solution to 10% sucrose in PB and shake gently for 30 min to 1 h.
  c. Exchange solution to 15% sucrose in PB and shake gently for 30 min to 1 h.
  d. Exchange solution to 20% sucrose in PB and shake gently for 30 min to 1 h.
  e. Freeze samples with OCT compound.

**Pause Point:** After taking images (step 13), samples were preserved in CUBIC-R (N). However, leaving samples in CUBIC-R (N) might induce signal dropping. To preserve fluorescent signals, it is better to keep samples in PBS at 4°C after PBS wash to remove CUBIC-R (N).

**Table:** Details of LSFM

Fluorescent protein/dyes	Excitation laser	Emission filter
FITC, GFP, Azami Green	488 nm	495–540 nm, Φ 32 mm
mCherry, Alexa Fluor 594	590 nm	610–640 nm, Φ 32 mm
RedDot2, Alexa Fluor 647	639 nm	660–750 nm, Φ 32 mm
tdTomato, Kusabira Orange	532 nm	590–650 nm, Φ 25 mm

## Expected Outcomes

**Figure 2:**
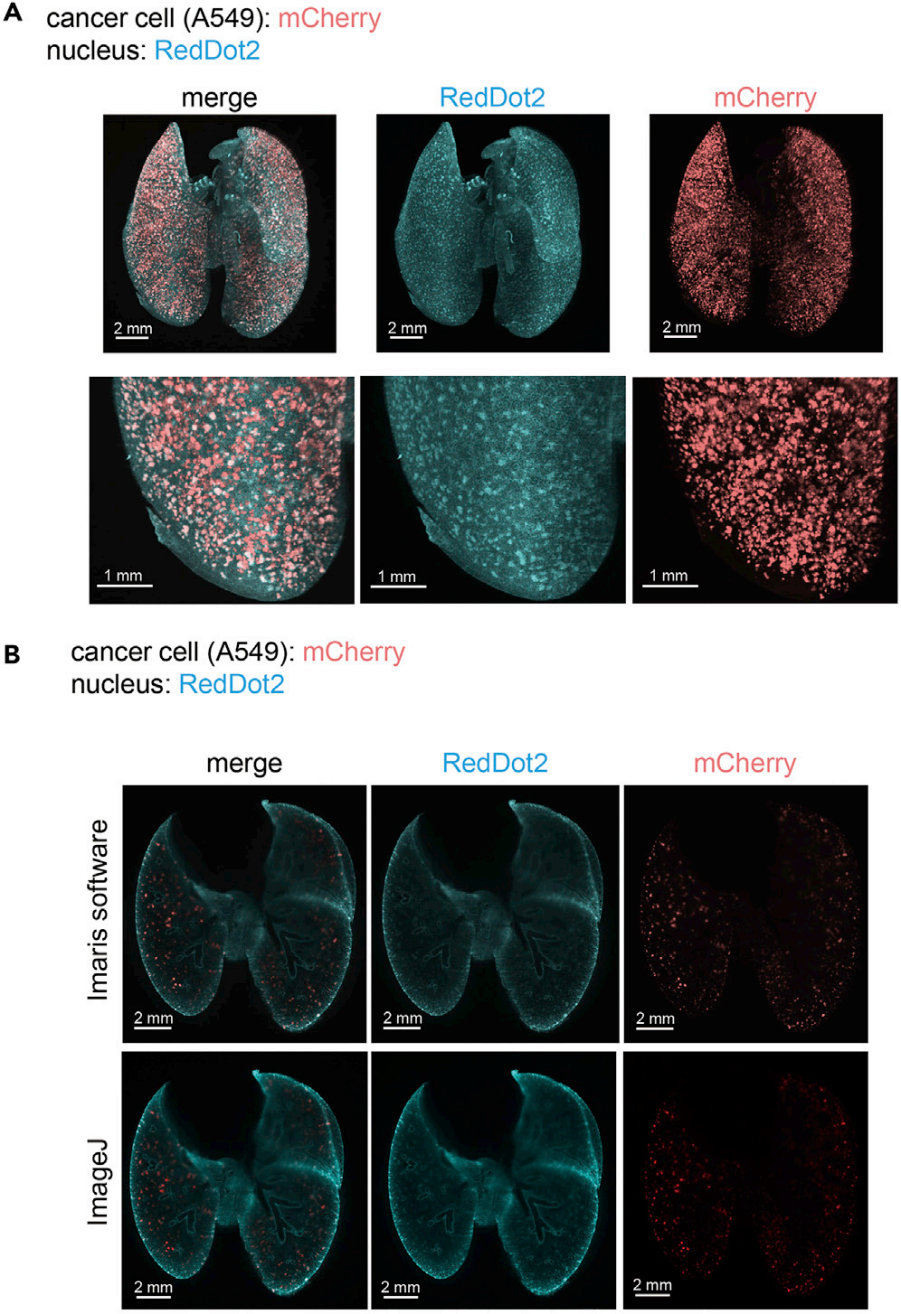
Whole Organ Imaging with the Experimental Lung Metastasis Model A549-mCherry-Luc2 cells were pre-treated with TGF-β1 for 2 days. These cells were injected intravenously (iv) into nude mice. Ten days after iv injection, mice were sacrificed and excised lungs were subjected to CUBIC procedures. The 3D images (A) and 2D images (B) were analyzed using Imaris software (A), (B) or ImageJ (B).

**Figure 3:**
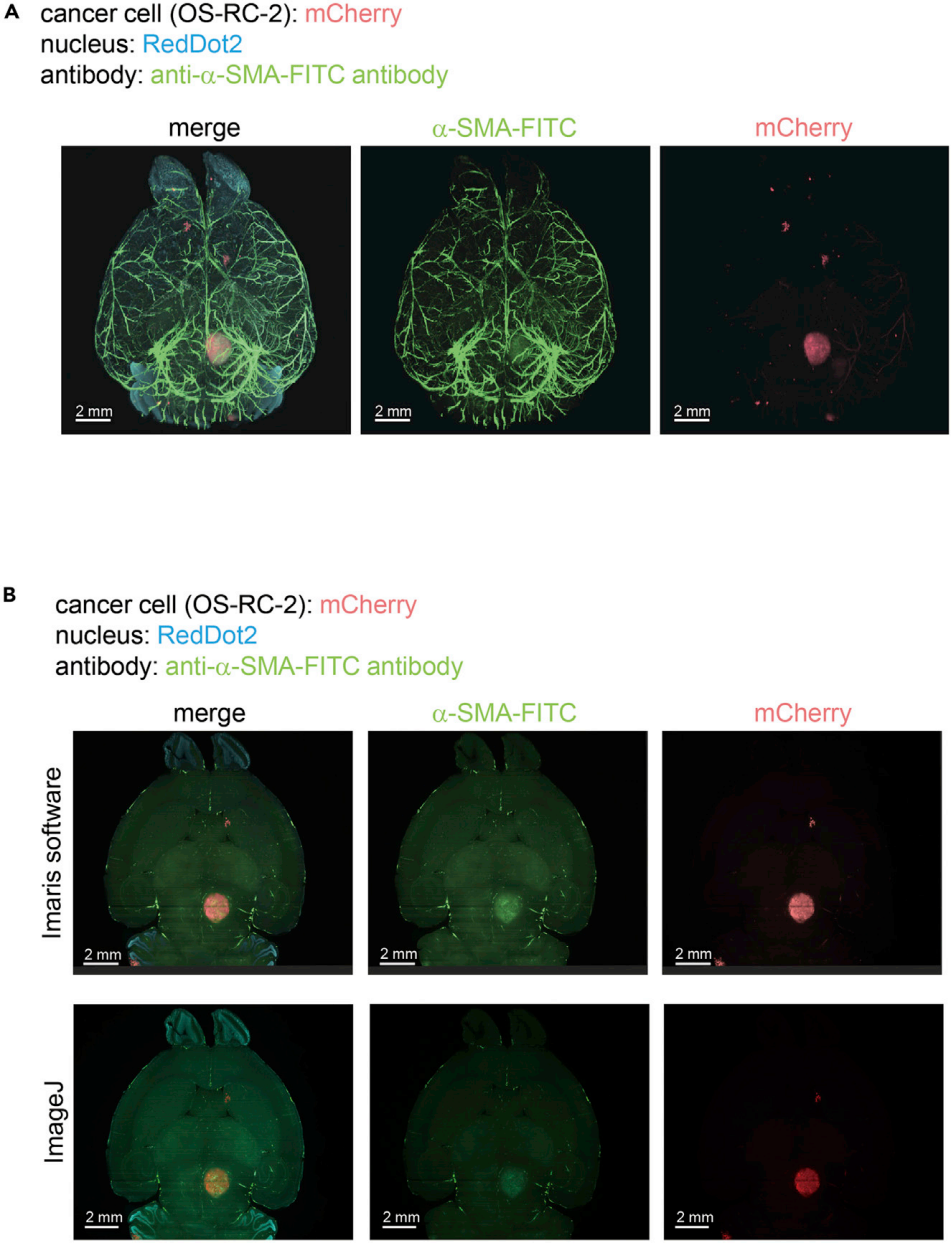
Whole Organ Imaging with the Experimental Brain Metastasis Model OS-RC-2-mCherry-Luc2 cells were injected intracardially (ic) into nude mice. Forty days after ic injection, mice were sacrificed and excised brains were subjected to CUBIC procedures. Brains were stained with α-SMA antibody conjugated FITC and RedDot2. The 3D images (A) and 2D images (B) were analyzed using Imaris software (A), (B) or ImageJ (B).

**Figure 4:**
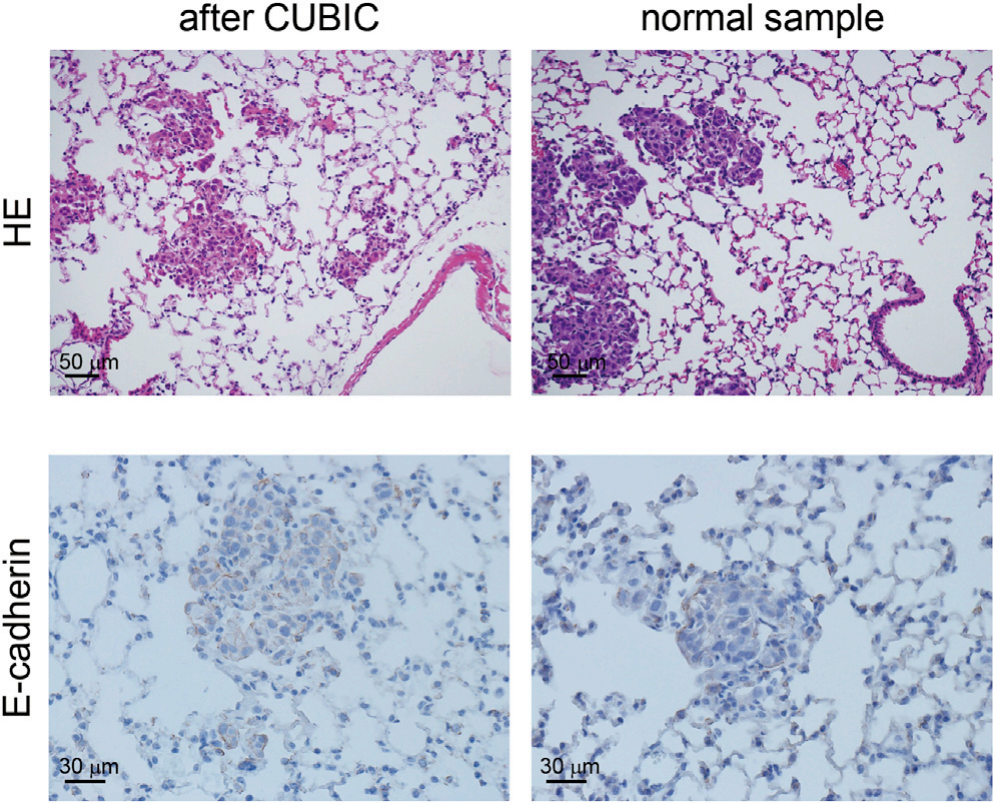
Immunohistochemistry after CUBIC Procedures Transparent lung samples, shown in Figure 2, were washed with PBS and processed for paraffin embedding. H&E staining and immunohistochemistry of E-cadherin (ABC method) was conducted (left, paraffin-embedded samples after CUBIC procedures; right, paraffin-embedded normal samples without CUBIC procedures).

Using this protocol, we can make various organs from mice transparent with high quality (Figures 1C and 1D). With these transparent mouse organs, we can capture multiple 2D images without sectioning. Reconstitution of these 2D images makes us possible to get 3D images of the intact mouse organs. For example, A549 cells expressing mCherry pre-treated with TGF-β were inoculated intravenously and lungs were excised on day 10. After CUBIC procedures, cancer metastases were observed in deep tissue (Figure 2, Methods Video S1). OS-RC-2 cells expressing mCherry were inoculated intracardially and brains were excised on day 40. Using CUBIC procedures, cancer metastasis and α-SMA positive mature blood vessels in brain were observed (Figure 3, Methods Video S2). When CUBIC-cleared organs were washed with buffer, they can be processed for standard paraffin-embedded histological tissue processing and analysis. Post-H&E staining or post-immunohistochemistry is useful for further investigation (Figure 4).

Methods Video S1. Whole Organ Imaging with Experimental Lung Metastasis Model, (Related to Expected Outcomes) The result of Figure 2 is shown as a movie, analyzed by Imaris software.

Methods Video S2. Whole Organ Imaging with Experimental Brain Metastasis Model, (Related to Expected Outcomes) The result of Figure 3 is shown as a movie, analyzed by Imaris software.

## Quantification and Statistical Analysis

1. Make tiff files to Imaris file by using Imaris File Converter. It is installed automatically when you install Imaris or Imaris Stitcher or you can download a free version.
2. Analyze Imaris file with Imaris software or analyze tiff files using Image J (Figures 2 and 3).
3. To evaluate the volume or the number of metastatic colonies with Imaris software, add new surface and decide threshold. The data is outputted as csv file.

## Limitations

Autofluorescence sometimes becomes a problem, especially with green fluorescence proteins such as green fluorescent protein (GFP). When signals from cancer cells are low, autofluorescence can mask the positive signals. In our current protocol, delipidation process is essential and structure with lipid was damaged and disappeared. Various pigments such as heme can be removed with CUBIC-L; however, some pigments such as melanin are not removed with CUBIC-L (Ueda et al., 2020). Moreover, CUBIC-R (N) reagent is not compatible with Alexa 488 and Dylight 488.

Perfusion with PBS and PFA process is needed to reduce autofluorescence signals; therefore, cancer cells in blood vessels are hardly detected in our current protocol.

This protocol is useful for detecting cancer cells expressing fluorescence proteins in mice, not optimized to 3D staining. However, 3D staining protocol is currently optimized and various antibodies can be used in CUBIC (Susaki et al., 2020).

## Troubleshooting

### Problem 1

Mouse organs do not become transparent.

### Potential Solution

Increasing the temperature and the length of the tissue-clearing process, especially delipidation and decolorization process with CUBIC-L, improves the transparency of the samples, but might lead to a decrease in the signal.

### Problem 2

Signals from fluorescence proteins cannot be detected.

### Potential Solution

Check pH of CUBIC-L and CUBIC-R (N), because high or low pH can quench the signals from fluorescence proteins. To enhance the signals from fluorescence protein, immunostaining with antibody (i.e., anti-GFP antibody) should be done. It makes us possible to monitor the signals from quenched fluorescent signals.

### Problem 3

3D staining does not work.

### Potential Solution

Although depending on the antibodies, post-fixation of the stained organs after step 9 with PFA (4°C–22°C, 1–16 h, depending on samples) might improve the staining efficiency.

## Resource Availability

### Lead Contact

Further information and requests for resources and reagents should be directed to and will be fulfilled by the Lead Contact, Kohei Miyazono (miyazono@m.u-tokyo.ac.jp) and Hiroki R. Ueda (uedah-tky@umin.ac.jp).

### Materials Availability

This study did not generate new unique materials.

### Data and Code Availability

The data of this study are available from the corresponding authors upon reasonable request.
